# Trace elements in the muscle and liver tissues of *Garra shamal* from the freshwater ecosystem of Oman: an exposure risk assessment

**DOI:** 10.1007/s11356-024-32229-w

**Published:** 2024-01-30

**Authors:** Saud M. Al Jufaili, Milad Adel, Seyed Pezhman Hosseini Shekarabi, Chiara Copat, Josef Velisek

**Affiliations:** 1 Department of Marine Science and Fisheries, Sultan Qaboos University, Al Khod 123, P.O Box 34, Muscat, Oman; 2 Agricultural Research, Education and Extension Organization (AREEO), Iranian Fisheries Science Research Institute (IFSRI), Tehran, Iran; 3 Agricultural Research, Education and Extension Organization (AREEO), National Research Center of Saltwater Aquatic Animals, Iranian Fisheries Science Research Institute (IFSRI), Bafq, Iran; 4 Department of Medical Sciences, Surgical and Advanced Technologies “G.F. Ingrassia”- Hygiene and Public Health, Laboratory of Environmental and Food Hygiene (LIAA), University of Catania, Via S. Sofia, 87, 95123 Catania, Italy; 5 Faculty of Fisheries and Protection of Waters, South Bohemian Research Center of Aquaculture and Biodiversity of Hydrocenoses, Research Institute of Fish Culture and Hydrobiology Vodnany, University of South Bohemia Ceske, Budejovice, Czech Republic

**Keywords:** Metals, Bioaccumulation, Fish, Risk assessment, Oman

## Abstract

Anthropogenic activities lead to environmental contamination with foreign substances such as heavy metals. This work was aimed to monitor trace elements (total arsenic (As), cadmium (Cd), chrome (Cr), cobalt (Co), copper (Cu), lead (Pb), manganese (Mn), mercury (Hg), nickel (Ni), and zinc (Zn)) contamination levels (dry weight base) in three natural freshwater reservoirs of Oman including Al Khawd and Al Amarat (Muscat Governorate) and Surur area (Ad Dakhiliyah Governorate as control area) using a native benthic inland fish (*Garra shamal*; Cyprinidae) for the first time. The muscle and liver of a hundred and twenty *G*. *shamal* were collected to assess the degree of metal contamination. Atomic absorption spectrometry was used as an analytical technique. From the spectrum of analyzed elements, we found Zn as a major element in monitored areas. The statistically significant (*P* < 0.05) highest concentrations of Zn liver (0.275 ± 0.065 µg/g) were in Al Amarat compared to the other areas. The concentrations of monitored elements in the fish muscle were lower than the liver samples. Furthermore, the fish length was significantly correlated with the accumulation of Hg and Co in both muscle and liver samples. In all analyzed fish from Oman inland water, the concentrations of elements were below the permissible limits; however, additional research is needed.

**Supplementary Information:**

The online version contains supplementary material available at 10.1007/s11356-024-32229-w.

## Introduction

Environmental pollution with metals has become a global threat, and the chief sources of these pollutants are mostly observed in areas with high levels of landfilling, mining and manufacturing, traffic and combustion by-products from fossil fuels, untreated sewage sludge, agricultural chemicals, and metal plating industries (Budi et al. 2022). Xenobiotics, such as metals, remain in the environment for a long time because of their poor degradability. Trace elements tend to accumulate in the muscle of aquatic animals and finally move upward through food chains and food webs (Sharma et al. 2023). Trophic transfer of trace elements can bring serious impacts on public health such as carcinogenic effects, several chronic disorders, growth demolition, physiological dysfunctions, and genetic-behavioral abnormalities in humans (Engwa et al. 2019; Nguyen and Kim 2023; Triassi et al. 2023). In recent decades, assessment of heavy metals in aquatic ecosystems has been the core of attention due to the huge release of heavy metals into water bodies through anthropogenic and natural resources.

In general, heavy metals are considered as trace elements because of their presence in minute concentrations. Heavy metals have a relatively high density, and some are essential for cell functions and toxic in high concentrations (known also as essential trace elements). However, others have no biological role (non-essential trace elements) and have a greater degree of toxicity even at very low concentrations which are well-known as toxic metals. Heavy metal poisoning can be caused by consuming the contaminated resources directly or through the food chain. Among the heavy metals, lead (Pb), mercury (Hg), and cadmium (Cd) are not only non-essential for living organisms but they are highly poisonous and can create serious health problems (Kumari and Mishra 2021). For instance, Cd is commonly leaked to the environment by using high-phosphate fertilizers and manufacturing batteries. This element does not have any physiological role in the human body and causes serious biological dysfunctions even at a low concentration (Robards and Worsfold 1991). Also, arsenic (As) is mainly emissions from acid mine drainage; As-bearing minerals, dye and glass industries in the world, and long-term exposure to even low levels of As can increase the risk of skin and lung cancers (Shams et al. 2022). The presence of a very small level of nickel (Ni) in food is necessary for the body, but when present in a high concentration, it is lethal by disrupting cardiovascular, respiratory, and nervous system functions (Macomber and Hausinger 2011). However, zinc (Zn), copper (Cu), manganese (Mn), iron, and selenium are necessary to maintain the metabolism of the human body as important macronutrient examples, but excessive consumption can be toxic (Godswill et al. 2020). Therefore, assessment of heavy metals is a key tool for protecting public health and the environment.

Oman is located in southwest Asia with a dry and warm climate. The country has several freshwater resources named wadis (shallow watercourses) with a great number of inland fish species belonging to Cypriniformes, Cyprinodontiformes, and Gobiiformes (approximately 80% of the total inland fish) (Esmaeili et al. 2022). Freshwater fish, living in a closed environment, are more susceptible to heavy metal accumulation than marine fish due to the differences in the osmoregulation process caused by salt loss and water uptake (Mensoor and Said 2018), and they are more vulnerable to industrial and domestic wastewater contaminations. Despite the high bioaccumulation potential of metals in freshwater aquatic animals, there is no report on the assessment of metals in freshwater fish species from the inland water ecosystem of Oman. Therefore, in this project, some of the candidate trace elements namely As, Cd, Cr, Co, Cu, Pb, Mn, Hg, Ni, and Zn were determined in the most abundant native benthic freshwater fish, *Garra shamal* (Teleostei: Cyprinidae), in three different areas in Oman for the first time. This study was also conducted to find out whether oral consumption of this fish species may pose a risk to human health, considering the average daily intake (EDI) and the target hazard quotient (THQ).

## Material and methods

### Sampling and study area

**Fig. 1:**
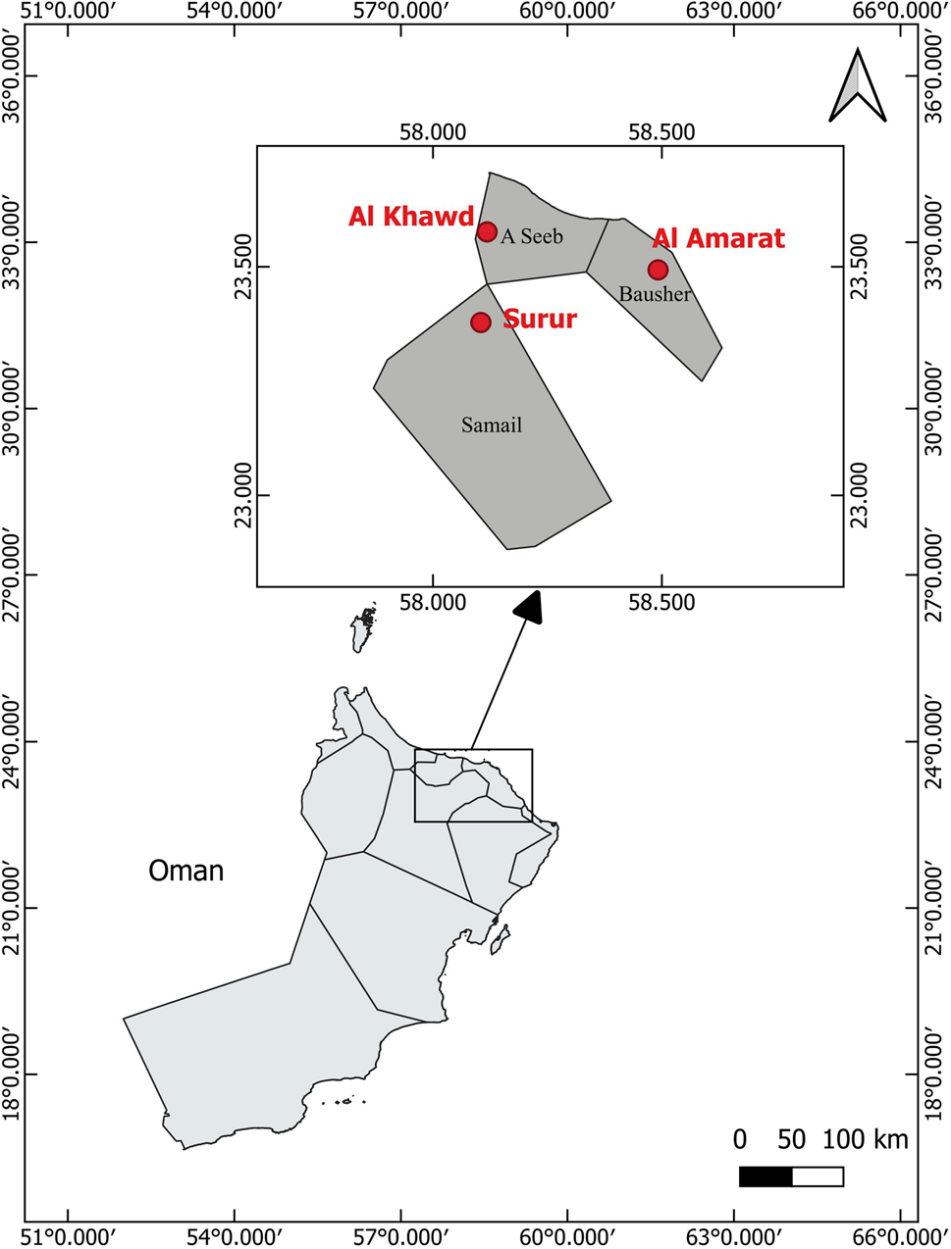
Location of the studies area and sampling areas

One hundred and twenty specimens of *G. shamal* (Teleostei: Cyprinidae) with a body weight of 1.92 ± 1.37 g (mean ± standard deviation) and a length of 47.4 ± 13.9 mm were collected between November and December 2022 with a trap net in Al Amarat, Al Khawd, and Surur areas (*n* = 40 in each location). The sampling areas were located in three different parts of Oman (Fig. 1): Al Khawd (23° 29′ 59″ N, 58° 29′ 52″ E), Al Amarat (23° 37′ 26″ N, 58° 33′ 45″ E), and Surur (23° 22′ 41″ N, 58° 6′17″ E). In this research, the candidate areas included the Al Amarat and Al Khawd regions, which are located near industrial areas with various potential pollutants (*i.e.,* metal hydraulic repair service, signs and banners manufacturing, metal scrap, plastic injection molding factory, pumping and pipelines manufacturers, aluminum window producer, steel furniture factory, and medical waste treatments). Besides, the Surur region was selected as the control area, because it is far from industrial and human activities and has clean freshwater resources. The fish samples were placed in the coded plastic bags and immediately transferred to the laboratory with ice. The total length of sampled fish was measured instead of body weight to find the relationship between the body size and heavy metal accumulation to avoid the influence of stomach contents. The specimens were then dissected to collect the livers and muscle samples, and all samples were coded and stored at − 20 °C for further analysis for less than 1 month.

### Chemical analyses

The procedure used to measure trace element concentrations in the fish samples was described previously (Adel et al. 2016). Trace element concentrations were measured in the muscle and liver tissue samples based on the standard methods with a minor modification. The muscle or liver tissues were oven-dried until constant weights were obtained. Edible aliquots of muscle or liver were accurately weighed and digested by high-pressure decomposition vessels according to the method described in our previous study (Dadar et al. 2014). A sample mixed with 4 mL of 68% nitric acid (Suprapur; Romil Ltd., Cambridge, UK), 4 mL of 30% hydrogen peroxide (Suprapur; Merck, Darmstadt, Germany), and 1 mL concentrated perchloric acid (Suprapur; Merck, Darmstadt, Germany). For Hg digestion, 45 mg V_2_O_5_ was added to the samples. Then, they diluted to 50 mL with 20 mL of distilled water and K_2_Cr_2_O_7_ (2%). The digestion process was conducted on a hotplate at 90 °C for 3 h or until clear, and all particles had turned a white color. The digested samples were filtered through a 0.45-mm membrane filter of nitrocellulose, diluted with high purity deionized water at a ratio of 1:5 and analyzed with flame atomic absorption spectrophotometry (Thermo M5 Series AA, Germany) equipped with a microcomputer-controlled acetylene flame. In addition, determination of total mercury was performed by the cold vapor hydride generation atomic absorption spectrometer (AAS 400, FIAS 100, PerkinElmer). In this survey, we use spikes for Mn and Co, and DORM-3 for the other elements. The overall recovery rates (mean ± SD) of Zn, Cu, Cr, Ni, Hg, Cd, Pb, As, Mn, and Co were 90 ± 3.3%, 95 ± 3.9%, 90 ± 3.2%, 94 ± 9.6%, 90 ± 2.6%, 90 ± 12.4%, 88 ± 8.1%, 98 ± 6.4%, 84 ± 4.2%, and 88 ± 4.3%, respectively. The limit of detection (LOD) of Zn, Cu, Cr, Ni, Hg, Cd, Pb, Co, As, and Mn was 0.05 ± 0.01, 0.01 ± 0.005, 0.005 ± 0.001, 0.005 ± 0.001, 0.001 ± 0.0001, 0.001 ± 0.001, 0.005 ± 0.001, 0.01 ± 0.005, 0.003 ± 0.001, and 0.002 ± 0.001 µg/g, respectively. The samples were analyzed in triplicate, and the results were collected on a dry-weight basis. Blanks are processed in the same way as the samples.

### Public health risk evaluation

The first step in risk analysis was to compare the estimated daily intake per meal size (EDIm µg/Kg b.w) with the reference dose (RfDo), by assuming a standard meal of 227 g for the adult population (US-EPA 2000).

Secondly, according to the real average freshwater consumption of the Oman population, calculated by FAOSTAT in 2020 (0.49 kg/capita/day) (FAOSTAT 2023; https://www.fao.org/faostat/en/#data/FBS), we have calculated the THQ, to understand if the food can pose a risk to human health during the lifetime. According to US-EPA, a THQ value below 1 means that the level of exposure is smaller than the reference dose, which assumes that daily exposure at this level is not likely to cause any pernicious effects during lifetime for the human population.

The estimated daily intake per meal size (EDIm) and target hazard quotient (THQ—unitless) were calculated according to the equation reported in previous reports (Conte et al. 2015; Adel et al. 2022; Giovis et al. 2022) as follows:

$$\begin{array}{c}{\text{EDIm}}={\text{MS}}\times {\text{C}}/{\text{BW}}\\ {\text{THQ}}=\left({\text{EF}}\times {\text{ED}}\times {\text{MS}}\times {\text{C}}\right) / \left({\text{RfD}}\times {\text{BW}}\times {\text{AT}}\right)\end{array}$$

where MS is the adult meal size (expressed as 227 g in EDIm), *C* is the metal concentration in the muscle tissue (expressed as µg/g wet weight), BW is the body weight (adults, 70 kg), IR is the average annual ingestion rate of freshwater fish for Oman population (0.0013 kg/capita/day), EF is the exposure frequency or number of exposure events per year of exposure (350 days/year), ED is the exposure duration (adults, 70 years), RfDo is the oral reference dose (mg/Kg/day) set by US EPA (2000), and AT is the averaging time (it is equal to EF × ED).

The EDIm and THQ value for As was calculated assuming the inorganic form as 3% of the total (Ferrante et al. 2019; Salvaggio et al. 2019) and methyl-mercury (CH_3_Hg) as 100% of the total (Salvaggio et al. 2019).

Furthermore, we have calculated the metal pollution index (MPI) in both the muscle and liver, which is a valuable mathematical model that summarize the value of all metals in a single form, and it is applied to evaluate metal in the food and the aquatic ecosystem. The MPI was calculated according to the following equation, where Cf is the concentration of each element (Copat et al. 2012):

$$\mathrm{MPI\; \mu g}/{\text{g}}=\left({\text{Cf}}1\times {\text{Cf}}2\times {\text{Cf}}2\times \dots ..\times {\text{Cfn}}\right)1/{\text{n}}$$

### Statistical analysis

Kolmogorov–Smirnov and Bartlett’s tests were applied to assess normal distribution data and the homoscedasticity of variance, respectively. Two-way ANOVA with Tukey’s post hoc test was used to determine whether the results of treatments (different tissues—muscle and liver) of the fish were significantly different among monitored areas (*P* < 0.05). All values were expressed as means ± SD. For comparison between concentrations of trace elements in liver and muscle, we used two-way ANOVA followed by Tukey’s post hoc test for multiple comparisons. Analysis was performed using STATISTICA v. 12.0 for Windows (STATSOFT, Inc.). In addition, the relationships among the heavy metals and the fish total length of all analyzed samples were performed by the Pearson’s correlation using R software v4.2.2.

## Results

### Trace elements

The concentrations of metals in *G*. *shamal* muscle and liver at the monitored areas are presented in Table 1. The least polluted area for all analyzed elements was Surur station. From the spectrum of analyzed trace elements, we found high levels of Zn in the monitored areas. The statistically significant (*F* = 8.21, *P* = 0.004) highest concentration of Zn in the liver (0.275 ± 0.064 µg/g) was in Al Amarat compared to the other areas.

**Table 1:** Concentrations of trace elements measured in the muscle and liver of *Garra shamal* in monitored sites, MPI index, and maximum levels set by regulatory agency

Locality	Maximum levels for metals in muscle meat of fish (µg/g wet weight)			Muscle (µg/g wet weight, mean ± SD, min–max)			Liver (µg/g wet weight, mean ± SD, min–max)		
	EC No. 915/2023	FAO 66/2003	CODEX STAN 193–1995	Al Amarat	Al Khawd	Surur	Al Amarat	Al Khawd	Surur
As	-	-	0.1*	0.013 ± 0.010^aA^ 0.005–0.040	0.008 ± 0.003^aA^ 0.003–0.012	0.017 ± 0.016^aA^ 0.005–0.060	0.059 ± 0.031^abB^ 0.010–0.090	0.101 ± 0.096^bB^ 0.020–0.400	0.031 ± 0.018^aA^ 0.008–0.070
Cd	0.05	0.05	-	0.008 ± 0.002^aA^ 0.004–0.012	0.021 ± 0.011^bA^ 0.008–0.040	0.007 ± 0.002^aA^ 0.001–0.010	0.022 ± 0.013^aB^ 0.010–0.050	0.049 ± 0.022^bB^ 0.010–0.080	0.028 ± 0.013^aB^ 0.010–0.060
Cr	-	-	-	0.029 ± 0.016^aA^ 0.010–0.070	0.051 ± 0.028^bA^ 0.010–0.090	0.016 ± 0.006^aA^ 0.010–0.030	0.038 ± 0.026^bA^ 0.010–0.080	0.050 ± 0.019^bA^ 0.020–0.090	0.013 ± 0.005^aA^ 0.006–0.020
Co	-	-	-	0.050 ± 0.018^bA^ 0.030–0.080	0.050 ± 0.017^bA^ 0.020–0.080	0.020 ± 0.010^aA^ 0.010–0.040	0.094 ± 0.037^aB^ 0.030–0.170	0.070 ± 0.026^aA^ 0.030–0.120	0.071 ± 0.020^aB^ 0.030–0.100
Cu	-	-	-	0.052 ± 0.022^aA^ 0.020–0.100	0.152 ± 0.049^bB^ 0.060–0.230	0.058 ± 0.031^aA^ 0.010–0.120	0.091 ± 0.046^bB^ 0.030–0.185	0.050 ± 0.019^aA^ 0.020–0.080	0.058 ± 0.023^aA^ 0.020–0.090
Pb	0.3	0.2	0.3	0.021 ± 0.010^aA^ 0.010–0.040	0.060 ± 0.019^bA^ 0.030–0.090	0.014 ± 0.006^aA^ 0.010–0.030	0.082 ± 0.025^bB^ 0.040–0.130	0.159 ± 0.036^cB^ 0.080–0.230	0.020 ± 0.012^aA^ 0.007–0.040
Mn	-	-	-	0.051 ± 0.025^bA^ 0.010–0.090	0.040 ± 0.021^abA^ 0.008–0.080	0.025 ± 0.014^aA^ 0.009–0.060	0.092 ± 0.042^bB^ 0.040–0.180	0.073 ± 0.027^abB^ 0.040–0.120	0.050 ± 0.028^aB^ 0.010–0.100
Hg	0.5	0.5	-	0.009 ± 0.009^bA^ 0.006–0.014	0.004 ± 0.002^aA^ 0.001–0.009	0.002 ± 0.001^aA^ 0.001–0.004	0.010 ± 0.002^cA^ 0.005–0.013	0.005 ± 0.002^bA^ 0.002–0.009	0.003 ± 0.001^aB^ 0.001–0.004
Ni	-	-	-	0.082 ± 0.034^bA^ 0.010–0.150	0.050 ± 0.027^abA^ 0.010–0.100	0.041 ± 0.031^aA^ 0.006–0.100	0.102 ± 0.038^aA^ 0.040–0.170	0.080 ± 0.009^aB^ 0.060–0.090	0.080 ± 0.025^aB^ 0.050–0.140
Zn	-	-	-	0.218 ± 0.06^aA^ 0.130–0.310	0.194 ± 0.05^aB^ 0.130–0.320	0.196 ± 0.070^aB^ 0.080–0.350	0.275 ± 0.064^bB^ 0.130–0.340	0.141 ± 0.039^aA^ 0.080–0.210	0.106 ± 0.029^aA^ 0.050–0.160
MPI	-	-		0.033	0.038	0.020	0.062	0.059	0.032

Values with different small letters in superscripts are significantly (*P* < 0.05) different among the locality groups. Values with different capital letters in superscripts are significantly (*P* < 0.05) different among the tissue groups in one locality

^*^Edible fat and oil

Significant higher levels of Mn (*F* = 6.31, *P* = 0.008), Hg (*F* = 6.98 *P* = 0.007), and Ni (*F* = 9.11, *P* = 0.003) were obtained in the muscle of *G*. *shamal* in Al Amarat compared to the other monitored areas. The significantly highest Cd (*F* = 7.61, *P* = 0.005), Cr (*F* = 9.44, *P* = 0.004), and Pb (*F* = 6.83, P = 0.007) contents were recorded in the muscle of *G*. *shamal* in Al Khawd compared to the other areas. Co level was significantly (*F* = 8.64, *P* = 0.003) higher in the muscle of *G*. *shamal* in both Al Amarat and Al Khawd areas compared to the Surur area. The As (*F* = 1.33, *P* = 0.115) and Zn (*F* = 1.87, *P* = 0.246) levels in the muscle samples were not significantly different between the monitored areas.

Significantly, (*P* < 0.05) higher Cu (*F* = 7.99, *P* = 0.003) and Zn (*F* = 9.68, *P* = 0.004) levels were in the liver of *G*. *shamal* in Al Amarat compared to the other monitored areas. Significantly higher (*F* = 9.54, *P* = 0.004) As and Cd levels were in the muscle of *G. shamal* in Al Khawd compared to the other areas. Significantly (*F* = 7.68, *P* = 0.005) higher Cr level was in the muscle of *G*. *shamal* in Al Amarat and Al Khawd compared to the Surur area. The Pb (*F* = 5.21, *P* = 0.010) and Hg (*F* = 5.99, *P* = 0.009) levels in the liver differed significantly between the monitored areas.

The Co (*F* = 1.67, *P* = 0.235) and Ni (*F* = 1.52, *P* = 0.284) levels in the liver were not significantly different among the monitored areas.

Significantly, higher concentrations of Cd (*F* = 7.38, *P* = 0.006) and Mn (*F* = 7.42, *P* = 0.006) were in the liver than in the muscle in all monitored areas. Significantly higher concentrations of As (*F* = 5.33, *P* = 0.010), Co (*F* = 5.48, *P* = 0.011), Cu (*F* = 8.69, *P* = 0.003), Pb (*F* = 10.22, *P* = 0.001), and Zn (*F* = 6.32, *P* = 0.008) were in the liver than in muscle in the Al Amarat area. In Al Khawd area, significantly higher concentrations of As (*F* = 9.91, *P* = 0.010), Pb (*F* = 6.94, *P* = 0.007), and Ni (*F* = 6.28, *P* = 0.008) were in the liver than in the muscle of *G*. *shamal*, but the concentrations of Cu (*F* = 8.12, *P* = 0.004) and Zn (*F* = 6.23, *P* = 0.008) were higher in the muscle than in the liver. In Surur area, significantly, higher concentrations of Co (*F* = 8.33, *P* = 0.004), Hg (*F* = 7.42, *P* = 0.005), and Ni (*F* = 7.61, *P* = 0.005) were in the liver than in the muscle, but the concentration of Zn (*F* = 5.31, *P* = 0.011) was higher in the muscle than in the liver.

### Correlation analysis

**Fig. 2:**
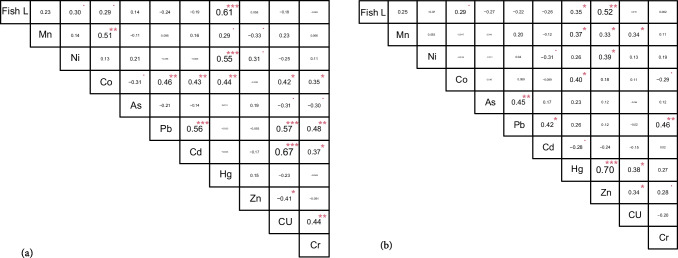
Pearson’s correlation between different heavy metals and total fish length (fish L) in *Garra shamal* muscle (**a**) and liver (**b**) samples. *P* ≥ 0.05, *p < 0.05, **P < 0.01, ***P < 0.001

As Fig. 2 reflects, positive correlations were found among Cd–Cu, Pb–Cu, Pb–Cd, and Ni–Hg in the muscle samples. The Pearson’s correlation coefficients between the heavy metals were 0.67, 0.57, 0.56, and 0.55, respectively (*p* < 0.001). Moreover, Hg (*r* = 0.61; *P* < 0.001), Ni (*r* = 0.30; *P* < 0.05), and Co (*r* = 0.29; *P* < 0.05) showed a significant positive correlation with the fish length in the muscle samples (Fig. 2a). In the liver samples, the results showed Hg was significantly correlated with Zn (*r* = 0.70; *P* < 0.001) (Fig. 2b). Furthermore, the fish length was significantly correlated with the accumulation of Zn (*r* = 0.52; *P* < 0.01), Hg (*r* = 0.35; *P* < 0.05), and Co in the liver samples (*r* = 0.29; *P* < 0.05) (Fig. 2b).

However, a significant negative correlation was recorded among Cu–Zn (*r* =  − 0.41), Cu–As (*r* =  − 0.31), Cr–As (*r* =  − 0.30), Co–As (*r* =  − 0.30), and Mn–Zn (*r* =  − 0.33) in the muscle samples (*p* < 0.05). In the liver samples, Cd–Ni (*r* =  − 0.31), Cd–Hg (r =  − 0.28), and Co–Cr (r =  − 0.29) were negatively correlated (*P* < 0.05).

### Public health risk evaluation

Results obtained for EDIm and THQ are shown in Table 2. They indicate that the Oman population have no risk to consume the selected fish species. As regard to EDIm, the estimated daily intake by assuming a real meal of 227 g for the adult population is several folds lower than the acceptable RfD, considered as the maximum acceptable oral dose of a toxic substance. Likewise, THQ is several folds below one. It was calculated with the average ingestion rate of freshwater fish of the Oman population, which is very low according to the FAOSTAT database. The results indicate that the elements’ concentration and consumption habit is not likely to cause any pernicious effects during the lifetime for the human population.

**Table 2:** Estimated daily intake (EDI µg/kg b.w.) and target hazard quotients (THQ) for individual metals/metalloids calculated for adults

Sampling area	Trace elements	As	Cd	Cr	Co	Cu	Pb	Mn	Hg	Ni	Zn
	RfD µg/Kg bw	0.3	1	3	0.3	40	3.5	140	0.1	20	300
Al Amarat	MSC_A_ Kg	53.85	8.75	7.24	0.42	53.85	11.67	192.16	0.78	17.07	96.33
	EDI µg/Kg bw	0.0013	0.0259	0.0940	0.162	0.1686	0.0681	0.1654	0.0292	0.2659	0.7069
	THQ (unitless)	2.32 × 10^−5^	1.42 × 10^−3^	1.72 × 10^−4^	2.97E-03	2.32 × 10^−5^	9.35 × 10^−5^	6.49 × 10^−6^	1.60 × 10^−3^	7.30 × 10^−5^	1.29 × 10^−5^
Al Khawd	MSC_A_ Kg	87.50	3.33	4.12	0.42	18.42	4.08	245.00	1.75	28.00	108.25
	EDI µg/Kg bw	0.0008	0.068	0.165	0.162	0.493	0.195	0.130	0.013	0.162	0.629
	THQ (unitless)	1.42 × 10^−5^	3.74 × 10^−3^	3.03 × 10^−4^	2.97E-03	6.77 × 10^−5^	2.67 × 10^−4^	5.09 × 10^−6^	7.12 × 10^−4^	4.45 × 10^−5^	1.15 × 10^−5^
Surur	MSC_A_ Kg	41.18	10.00	13.13	1.05	48.28	17.50	392.00	3.50	34.15	107.14
	EDI µg/Kg bw	0.0017	0.023	0.052	0.065	0.188	0.045	0.081	0.006	0.133	0.636
	THQ (unitless)	3.03 × 10^−5^	1.25 × 10^−3^	9.50 × 10^−5^	1.19E-03	2.58 × 10^−5^	6.23 × 10^−5^	3.18 × 10^−6^	3.56 × 10^−4^	3.65 × 10^−5^	1.16 × 10^−5^

## Discussion

In today’s world, one of the basic concerns of human society is the entry of various pollutants into the food chain from water ecosystems. Fish are exposed to metals from feeds and wild habitats (Ikem and Garth 2022). In this regard, *G*. *shamal* is the most abundant native cyprinid fish species in Oman freshwaters, which is bottom-dwelling and inhabits mainly the northern hill streams of Oman (Al Jufaili et al. 2022). They are omnivorous and feed primarily on the bottom organic debris, small crustaceans, worms, and mosquito larvae. Despite the small size of the fish (they reach a total length of up to 8.0 cm), they are consumed by the local people. Therefore, monitoring heavy metal pollution in these local fish is valuable from the point of view of public health.

In our study, we found that Zn concentration was much higher than other metals in the fish muscle and liver samples from monitored areas. The concentration of Zn as an essential element is commonly higher than toxic and non-essential metals in aquatic animals due to the important roles of this element in the body such as involving in the structure of many enzymes (Baramaki Yazdi et al. 2012; Lall and Kaushik 2021). On the other hand, the rate of Zn excretion is very slow compared to its bioaccumulation rate, which causes the high accumulation rate of Zn in fish (Tekin-Özan and Kir 2005). In this study, the highest concentration of Zn was obtained in Al Amarat in both liver and muscle samples. However, in this study, Zn concentrations did not exceed the risk international standards set by the United Nations Food and Agriculture Organization, which considers 40 mg of zinc a day to be the upper limit dose for adults and 4 mg of zinc a day for infants under age 6 months. Zinc is an essential element for living organisms such as fish, but higher concentration can cause harmful effects on fish species such as osmoregulation disorder, cardiac respiratory rhythm malfunction, changes in the blood gases and acid-alkaline status, tissue hypoxia (Tort et al. 1982), and histopathological organ damages (Çelik et al. 2013). A higher content of Zn was reported from different organs (kidney, gills, liver, and muscle) of *Garra gotyla* both upstream and downstream of Panjkora River, Pakistan (Ullah et al. 2016). The levels of Pb, Cu, Cd, and Ni in the present study are much higher than those conducted in *G*. *gotyla* from River Panjkora (Ullah et al. 2016) because of higher heavy metal inputs from the industrial and municipal effluents in the study area. However, future research in the study area is necessary to compare the results obtained in the present study.

Despite there are several documents reporting heavy metals contamination in marine fish and seafood products in Oman, there is no report on the heavy metal accumulation in freshwater systems to compare with the obtained results. However, there are documents showing a high concentration of some toxic metals in the suburban areas of Oman due to human civilization (Yaghi and AbdulWahab 2004; Al-Shidi et al. 2022). In a previous study, Gümgüm et al. (1994) examined the accumulation of some heavy metals in *Cyprinion macrostomus* and *Garra rufa* from Tigris River (Turkey), and they found the high accumulation levels of Cu in *G. rufa* muscle (188 ± 5.8 µg/g dry weight) and liver (1102 ± 213.2 µg/g dry weight) samples, which are thousand times higher than the presented study in *G*. *shamal* from Oman inland waters due to a copper mine (Ergani Copper Plant) near to Tigris River. The highest accumulation of Cu was recorded in *G*. s*hamal* muscle samples collected from Al Khawd area compared to other areas and liver. A higher level of Cu in *G*. *shamal* muscle is caused by the metal industries in Al Khawd. Essential metals such as Zn, Cu, Ni, and Cr participate in several biochemical reactions for normal physiological functions in the body. In our study, we found higher levels of Zn and Ni as well as some toxic metals like Co and Hg in the muscle and liver specimens from Al Amarat area. Al Amarat is the fifth most populated city in Muscat and is near the Al-Atkia industrial area. It has some industrial activities such as metal hydraulic repair service, metal scrap suppliers, signs and banners manufacturing as well as sewage treatment. In this context, Al Raisi et al. (2014) reported the exceeding levels of Al, V, Cr, Mn, Co, Ni, Ba, Pb, and Fe leachate pools located behind the landfill in Al Amarat regarding the electronic and painting wastes as well as dumping of steel scrap in the landfills. Research by AL Touqi from the College of Engineering, Sultan Qaboos University, Oman pointed out that Al Amarat landfills had remarkably high concentrations of metals especially Cu, Cr, and Zn in 2008 (Aljuboury et al. 2019).

In this study, the strong and significant correlations among the heavy metals in both liver and muscle samples with similar results of the spatial matrix may depict similar anthropogenic effects such as sewage effluents, untreated industrial discharges, and domestic wastewaters (Asim and Nageswara Rao 2021), which are the main source of contaminants in the inland waters of Oman. In locations polluted with heavy metals, the metal concentrations in the liver were higher compared with muscle tissues (Maršálek et al. 2005). Metal increases more drastically in the liver than in the muscle of fish exposed to high contamination, changing the liver/muscle metal ratio (Abreu et al. 2000). However, this study showed that As was negatively correlated with Cu, Cr, and Co in the muscle samples. The lack of consistent results in terms of Zn and Cu concentrations in the liver and muscle samples can be attributed to the different rates of absorption, deposition, and excretion of Zn and Cu in different fish organs (Jezierska and Witeska 2006). The negatively correlated trace elements also indicate the possible multiple sources of pollutants in the studied area (Kumar et al. 2019). As and Cu might have different origins, since the chief contamination sources of As are agricultural industry (chemical fertilizers and pesticides) and timber treatment (He et al. 2005). But natural and anthropogenic resources (*i.e.,* mining, metal and electrical manufacturing, antifouling paints, and sewage water) can be responsible for increasing Cu inputs in the environment. The potential contamination sources of Cu, Cr, and Zn have been found in the study areas (Aljuboury et al. 2019). In the liver samples, a significant negative correlation was found among Cd–Ni, Cd–Hg, and Co–Cr. In this context, Cr pollutes the environment primarily from machinery manufacturing, smelting enterprises, petrochemical activities, and electroplating plants (Wang et al. 2022); however, Co mainly originated from artisanal cobalt mines and production of Co alloys in electric car batteries and gadgets. The largest source of Ni pollution is fossil fuel combustion (Wu et al. 2018). Moreover, industrial sewage (*e.g*., acetaldehyde and chlor-alkali plants), coal burning, municipal and sewage sludge incineration, and mining, especially gold mining, are the main sources of waterborne Hg emissions (Guney et al. 2020). The anthropogenic activities such as manufacturing batteries, high phosphate fertilizers, plastic stabilizers, and Cd-based waste incineration are major sources of Cd pollution (Pham 2020; Khan et al. 2022), which are all different from Hg and Ni origins.

According to the results, Hg, Ni, and Co in the muscle and Zn, Hg, and Co in the liver were positively correlated with the fish length. The accumulation of heavy metals in living organisms is controlled through the metal absorption rate and their metabolism rate relative to the body size (Maggi et al. 2009). Previous documents pointed out that fish body size can be considered a key indicator in the assessment of heavy metal accumulation (Dang and Wang 2012; Jisr et al. 2020). Notably, this study illustrated that Hg and Co levels increased with increasing body length of *G. shamal* in both muscle and liver samples. This can be explained by decreasing growth rate and the metals elimination rate in larger *G*. *shamal* than in smaller (Mao et al. 2021). Metals are taken up through the gills and skin or through the feeds (El-Moselhy et al. 2014). Therefore, the accumulation of some heavy metals can increase with increasing body size due to nutritional habits, the amount of heavy metal pollution discharged into the environment, and variable metabolic and absorption rates of metals in different organs. Studies have shown that the relationship between the accumulation of different metals in the fish body may be due to the competitive or biological effect of metal binders and transporters at the cellular level of different organs (Wright 1995). Therefore, it seems that the positive correlation between the fish length with Hg and Co in *G*. *shamal* muscle and liver samples is due to the high tendency of the metals to bind mainly to proteins (Man et al. 2019). In terms of Hg, methylmercury is efficiently distributed in muscle tissue as a protein-rich organ (Berntssen et al. 2004). Unlike the muscle, where Hg mostly exists in its organic form, Hg accumulates in liver mostly in its inorganic form. These explanations can justify high accumulation levels of Hg in these organs (muscle and liver) with the increase of *G*. *shamal* body length. Co participates in the structure of vitamin B12 and a remarkable positive correlation was reported between the levels of Co and vitamin B12 in liver (Huwait et al. 2015). Moreover, the accumulation of heavy metals including Co increases over exposure time in liver with the increase of body size due to the presence of high metalloprotein that plays a vital role in the regulation and elimination of metals (Scharf et al. 2014). On the other hand, there is a strong tendency of Co complexes towards basic amino acids (Arderne et al. 2020), which have a high amount in the fish muscle. Additional research is needed to elucidate the physiological reasons for the strong correlation of Hg and Co in liver and muscle organs with fish body size in the future.

Although the maximum tolerable limit (MTL) for trace elements in fish muscle was lower than that reported by the major international regulatory organizations, it must be emphasized that toxic metals such as As, Cd, Hg, and Pb can still cause serious health problems in humans, even at low concentrations, if ingested over a long period of time (Adel et al. 2022).

In all analyzed fish from Oman inland waters, the concentrations of elements were below the permissible limits, as those defined in Codex Alimentarius. With regard to EDIm, the estimated daily intake by assuming a real meal of 227 g for the adult population is several folds lower than the acceptable RfD, considering the maximum acceptable oral dose of a toxic substance. As far as Hg concentrations (RfD—0.1 µg/Kg bw) are concerned, the maximum recommended intake of fish flesh from the Al Amarat, Al Khawd, and Surur areas would be 15.0, 23.3, and 52.5 kg per person per month, respectively. In Al-Mughairi et al. (2013) survey, the Hg level in studied fish including fresh, frozen, canned, dried, and smoked fish in Oman except in a few samples was lower than the limit value for human consumption recommended by several health organizations. In a similar study, Al-Busaidi et al. (2011) reported that heavy metals concentration in economical marine fish species in several regions of Oman were in the range of international standards levels recommended by EC (2001), FAO (1983), and FDA (2003).

## Conclusions

This study revealed that *G*. *shamal* can be a good indicator of freshwater quality due to its bottom-dwelling behavior and wide distribution in the inland waters of Oman. The results also indicate that the elements concentration and consumption habit are not likely to cause any pernicious effects during lifetime for the local community and other people due to low metal pollution indexes. Generally, the concentrations of monitored elements in the fish muscle were lower than the liver samples. However, further investigations are highly recommended to better understand the heavy metal effects on aquatic biota in the freshwater ecosystem of Oman and whether they constitute a health hazard.

### Supplementary Information

Below is the link to the electronic supplementary material.

Supplementary file1 (XLSX 16 KB)

## Data Availability

The dataset used and/or analyzed during the current study is available from the corresponding author upon reasonable request.
